# Reaction lumping in metabolic networks for application with thermodynamic metabolic flux analysis

**DOI:** 10.1038/s41598-021-87643-8

**Published:** 2021-04-20

**Authors:** Lea Seep, Zahra Razaghi-Moghadam, Zoran Nikoloski

**Affiliations:** 1 Bioinformatics, Institute for Biochemistry and Biology, University of Potsdam, 14476 Potsdam, Germany; 2 Systems Biology and Mathematical Modeling, Max Planck Institute of Molecular Plant Physiology, 14476 Potsdam, Germany

**Keywords:** Computational biology and bioinformatics, Systems biology

## Abstract

Thermodynamic metabolic flux analysis (TMFA) can narrow down the space of steady-state flux distributions, but requires knowledge of the standard Gibbs free energy for the modelled reactions. The latter are often not available due to unknown Gibbs free energy change of formation $, {\Delta }_{f} G^{0}$, of metabolites. To optimize the usage of data on thermodynamics in constraining a model, reaction lumping has been proposed to eliminate metabolites with unknown ${\Delta }_{f} G^{0}$. However, the lumping procedure has not been formalized nor implemented for systematic identification of lumped reactions. Here, we propose, implement, and test a combined procedure for reaction lumping, applicable to genome-scale metabolic models. It is based on identification of groups of metabolites with unknown ${\Delta }_{f} G^{0}$ whose elimination can be conducted independently of the others via: (1) *group implementation*, aiming to eliminate an entire such group, and, if this is infeasible, (2) a *sequential implementation* to ensure that a maximal number of metabolites with unknown ${\Delta }_{f} G^{0}$ are eliminated. Our comparative analysis with genome-scale metabolic models of *Escherichia coli*, *Bacillus subtilis,* and *Homo sapiens* shows that the combined procedure provides an efficient means for systematic identification of lumped reactions. We also demonstrate that TMFA applied to models with reactions lumped according to the proposed procedure lead to more precise predictions in comparison to the original models. The provided implementation thus ensures the reproducibility of the findings and their application with standard TMFA.

## Introduction

Constraint-based modeling of genome-scale metabolic models have been used to identify patterns in steady-state flux distributions, pointing at design principles of metabolic networks^1–3^, and to design metabolic engineering strategies for manipulation of metabolic processes^4–6^. Moreover, approaches from the constraint-based modeling framework have been employed to integrate heterogeneous high-throughput data, including: gene expression levels^7^, proteome abundances^8,9^, and metabolite concentrations^10–15^.

Genome-scale metabolic models provide a mathematical representation of all documented biochemical reactions that interconvert nutrients from the environment into extracted products and biomass^16^. A metabolic network is represented by a stoichiometric matrix, $S$, with $m$ rows, representing metabolites, and $n$ columns, denoting reactions. The entries of the stoichiometric matrix describe the role of a metabolite in a given reaction, such that negative and positive entries indicate that the metabolite enters as a substrate and product of the reaction, respectively. Approaches in the constraint-based framework often invoke the steady-state assumption, whereby the concentrations of metabolites, expressed as linear combination of fluxes that contribute to their synthesis and degradation, do not change with time^17^. As a result, Flux Balance Analysis (FBA), as the prominent approach on which the constraint-based framework rests, can provide predictions about steady-state flux distributions with the assumption that the biological system optimizes a particular task (e.g. maximizing cellular growth)^17^. However, fluxes directly depend on the concentration of enzymes and concentration of metabolites, leading to scenarios in which predictions of FBA do not consider constraints due to metabolite concentrations.

One approach that accounts for the effect of metabolic concentrations on metabolic fluxes is Thermodynamic Metabolic Flux Analysis (TMFA)^11,18^. This approach introduces additional constraints to ensure that the resulting steady-state flux distribution respects the laws of thermodynamics, thus restricting the space of feasible flux distributions. More specifically, TMFA allows a flux through a reaction only if associated change of Gibbs free energy ${\Delta }G$ is negative^19^. The value of $\Delta G_{j}$ for each reaction $r_{j}$, $1 \le j \le n$, depends on concentrations of participating metabolites, $x_{j}$, $1 \le j \le m$, the respective stoichiometric coefficient $s_{ij}$, the standard Gibbs free energy ${\Delta }G_{j}^{0}$, as well as the universal gas constant ${\text{R}}$ and temperature ${\text{T}}$, as follows:

$$\Delta G_{j} = \Delta G_{j}^{0} + RT\ln \left( {\prod\limits_{{1 \le i \le m}} {x_{i}^{{s_{{ij}} }} } } \right).$$

We note that ${\Delta G}_{{\text{j}}}^{0}$ can be obtained from the standard Gibbs free energy of formation of metabolites, ${\Delta }_{f} G^{0}$, weighted by the respective stoichiometric coefficients with which the metabolites enter the reaction $r_{j}$:

$$\Delta G_{j}^{0} = \mathop \sum \limits_{1 \le i \le m} s_{ij} \Delta_{f} G_{i}^{0} .$$

This approach has been extended to consider the contribution of different chemical groups, termed group contribution method^20,21^, and later, the contribution of pseudoisomeric groups^22^. However, for many metabolites ${\Delta }_{f} G^{0}$ is neither experimentally determined, due to the large experimental efforts and availability of chemical standards needed^23^, nor can be estimated by using the group contribution methods and extensions thereof^22^. The group contribution method is reported to not be applicable in the case of organic–inorganic complexes as well as for a small number but often encountered organic substructures^21^. As a result, the standard Gibbs free energy for more than a third of reactions in the entire KEGG database are missing since ${\Delta }_{f} G^{0}$ for the included metabolites are not available^21,22^. Moreover, in the most recent version of the ModelSEED database, more than half of the included metabolites have unspecified ${\Delta }_{f} G^{0}$ values^24^.

To overcome the challenge in TMFA, Henry et al. introduced the idea of determining a linear combination of reactions with undetermined ${\Delta }G^{0}$, so-called lumping, to obtain reactions in which metabolites with unknown ${\Delta }_{f} G^{0}$ are eliminated, i.e. enter with stoichiometric coefficients of zero^19^. Hence, ${\Delta }G^{0}$ of the resulting lumped reaction is fully specified. However, besides the idea and the list of lumped reactions, the process of lumping was not further specified in the original study.

Let the reactions be partitioned into two classes, $J^{lumped}$, composed of all lumped reactions, and $J^{model}$, consisting of the reactions comprising the original model. Therefore, the lumped reactions are only introduced to impose more thermodynamic constraints, while the steady-state flux space remains unaltered (by solving $Sv = 0$ for reactions in $J^{model}$). Reactions whose lumping leads to a lumped reaction $r_{k}$ can only be thermodynamically feasible if the lumped reaction itself is feasible^18^. This is ensured by the following constraints:

1 $${\Delta G}_{{\text{k}}} \le \left( {1 - {\text{y}}_{{\text{k}}} } \right){\text{M}}$$

2 $$\mathop \sum \limits_{{{\text{j}} \in {\text{J}}^{{{\text{model}}}} }} {\upalpha }_{{{\text{kj}}}} {\text{z}}_{{\text{j}}} \le \left( {\mathop \sum \limits_{{{\text{j}} \in {\text{J}}^{{{\text{model}}}} }} {\upalpha }_{{{\text{kj}}}} } \right) - \left( {1 - {\text{y}}_{{\text{k}}} } \right)$$

3 $${\Delta G}_{{\text{k}}} = {\Delta G}_{{\text{k}}}^{0} + {\text{RT}}\left( {\mathop \sum \limits_{{1 \le {\text{i}} \le {\text{m}}}} {\text{s}}_{{{\text{ik}}}} {\text{ln}}\left( {{\text{x}}_{{\text{i}}} } \right)} \right)$$

4 $${\text{r}}_{{\text{k}}} \in {\text{J}}^{{{\text{lumped}}}} ,{\text{r}}_{{\text{j}}} \in {\text{J}}^{{{\text{model}}}} ,$$

where the binary variables $y_{k}$ and $z_{j}$ take the value 1 if the respective lumped reaction $r_{k}$ and the model reaction $r_{j}$ are thermodynamically feasible. Here, $\alpha$ denotes the linear combination of reactions which yield the lumped reaction and $M$ is a big constant. Note that when ${\text{y}}_{{\text{k}}} = 0$, i.e. the lumped reaction is not thermodynamically feasible, then $\sum\nolimits_{{{\text{j}} \in {\text{J}}^{{{\text{model}}}} }} {{\upalpha }_{{{\text{kj}}}} {\text{z}}_{{\text{j}}} } \le \left( {\sum\nolimits_{{{\text{j}} \in {\text{J}}^{{{\text{model}}}} }} {{\upalpha }_{{{\text{kj}}}} } } \right) - 1$, implying that at least one of the reactions forming the lumped reaction ${\text{r}}_{{\text{k}}}$ is inactive. Despite advances in applications of TMFA across different organisms and for various purposes, from estimation of realistic flux distributions^19,25^ to model reduction^26,27^, the procedure of reaction lumping has not been fully specified. We would like to note that the reaction lumping does not consider removal of reactions while retaining key functional properties, as applied in stoichiometric techniques for model reduction with application of thermodynamic constraints^27,28^ or without them^29,30^.

Here we introduce and precisely formulate an approach for reaction lumping and provide an efficient implementation that is applicable with genome-scale metabolic models. The proposed formulation of the approach identifies a maximal subset of metabolites that can be eliminated by lumping, leading to automation of this step in the application of the constraint-based approaches based on TMFA.

## Methods

The calculation of standard Gibbs free energy, $\Delta {G}^{0}$, of reactions is hindered by the presence of metabolites with unknown ${\Delta }_{f}{G}^{0}$ . Reaction lumping aims to identify a linear combination, $\alpha$, of reactions involving at least one metabolite with unknown ${\Delta }_{f}{G}^{0}$, such that the corresponding stoichiometric coefficient in the resulting lumped reaction is zero. As a result, $\Delta {G}^{0}$ of the lumped reaction can be calculated, thus facilitating the application of TMFA. The proposed approach considers the potentially intertwined relationship of metabolites with unknown ${\Delta }_{f}{G}^{0}$ within a metabolic network, and ensures that every lumped reaction that is eventually created includes only metabolites with known ${\Delta }_{f}{G}^{0}$.

### Lumping program

**Figure 1:**
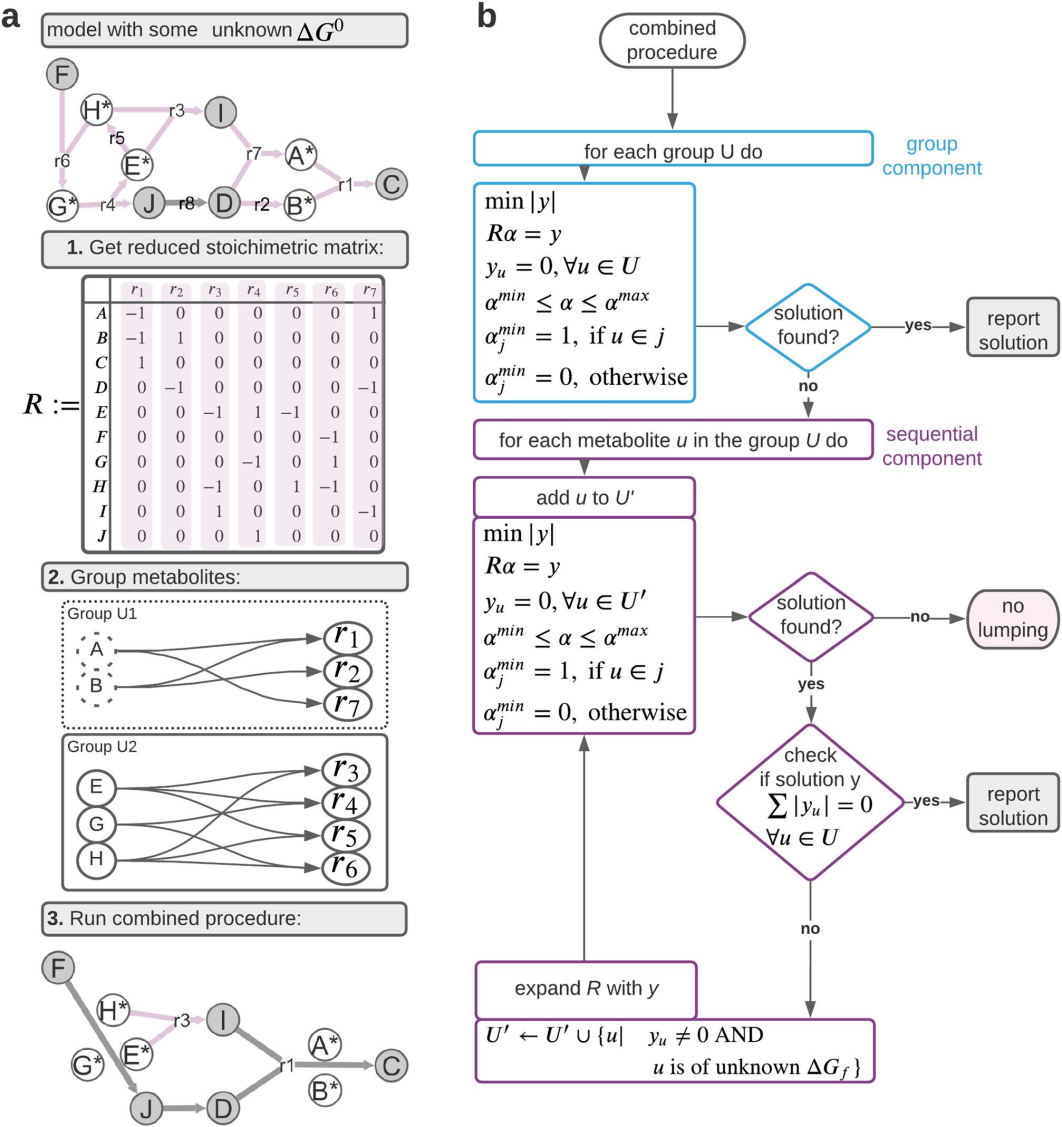
Reaction lumping workflow. Starting with a model involving reactions with unknown $\user2{\Delta G}^{0}$, a subset ${\varvec{R}}$ of the underlying stoichiometric matrix is extracted involving only those. Then the lumping procedure identifies linear combinations of reactions to arrive at a lumped reaction whose $\user2{\Delta G}^{0} \user2{ }$ can be calculated. (**a**) Proposed procedure identifies the reactions in ${\varvec{R}}$ (pink shaded) based on the involvement of metabolites without ${\varvec{\varDelta}}_{{\varvec{f}}} {\varvec{G}}^{0}$ (here marked with *). In a second step, all metabolites with unknown ${\varvec{\varDelta}}_{{\varvec{f}}} {\varvec{G}}^{0}$ are partitioned based on shared appearance in at least one reaction. A group is defined as connected component within a bipartite graph having metabolite with unknown ${\varvec{\varDelta}}_{{\varvec{f}}} {\varvec{G}}^{0}$. nodes and all reactions those metabolites participate in. Here, two groups, U1 and U2, are found. U1 consists of metabolites $\user2{A }$ and ${\varvec{B}}$, while U2 includes ${\varvec{G}},\user2{ }$ ${\varvec{E}}$ and ${\varvec{H}}$. (**b**) The proposed procedure tests if all metabolites within a group can be eliminated at once; if no solution can be found each metabolite in the group is evaluated on its own. Each lution here is preliminary; it must be checked whether there are any other metabolites in the group still involved (which would hinder the $\user2{\Delta G}^{0}$ calculation). If the latter hold true, such metabolites are added sequentially while each previous solution is placed in ${\varvec{R}}.$

Here, all reversible reactions are split into two irreversible reactions. If metabolites with unknown ${{\varvec{\Delta}}}_{{\varvec{f}}}{{\varvec{G}}}^{0}$ co-occur in the lumped reactions, a linear combination may not be able to eliminate all such metabolites of unknown ${{\varvec{\Delta}}}_{{\varvec{f}}}{{\varvec{G}}}^{0}$ at once, resulting in a lumped reaction for which ${\varvec{\Delta}}{{\varvec{G}}}^{0}$ can still not be calculated. To resolve this problem, we first define the notion of a group of metabolites with unknown ${{\varvec{\Delta}}}_{{\varvec{f}}}{{\varvec{G}}}^{0}$. We consider the submatrix *R* of the stoichiometric matrix ${\varvec{S}}$ that involves reactions whose ${\varvec{\Delta}}{{\varvec{G}}}^{0}$ cannot be determined due to the involvement of metabolites with unknown ${{\varvec{\Delta}}}_{{\varvec{f}}}{{\varvec{G}}}^{0}$ (Fig. 1a). These reactions can be represented by a bipartite graph composed of reaction and metabolite nodes. A metabolite node is connected to a reaction node if the corresponding metabolite participates in the reactions. To determine the groups of metabolites with unknown ${{\varvec{\Delta}}}_{{\varvec{f}}}{{\varvec{G}}}^{0}$, first the metabolites with available ${{\varvec{\Delta}}}_{{\varvec{f}}}{{\varvec{G}}}^{0}$ are removed from the bipartite graph. The connected components in the resulting graph correspond to the groups of metabolites with unknown ${{\varvec{\Delta}}}_{{\varvec{f}}}{{\varvec{G}}}^{0}$. For instance, in Fig. 1a, metabolite A and B, with unknown ${{\varvec{\Delta}}}_{{\varvec{f}}}{{\varvec{G}}}^{0}$ form a group since no other metabolites of unknown ${{\varvec{\Delta}}}_{{\varvec{f}}}{{\varvec{G}}}^{0}$ participate in the reactions ${{\varvec{r}}}_{1}$, ${{\varvec{r}}}_{2}$ and ${{\varvec{r}}}_{7}$, that include these metabolites.

The resulting lumped reaction for a group, $U$, of metabolites with unknown ${\Delta }_{f} G^{0}$ is given by the product $y = R\alpha$ in which the stoichiometry of each metabolite $u \in U$, specified with ${\text{y}}_{{\text{u}}}$, is constrained to 0:

5 $$\mathop {{\text{min}}}\limits_{{{\upalpha },{\text{y}}^{ + } ,{\text{y}}^{ - } }} \sum {\text{y}}^{ + } + {\text{y}}^{ - }$$

6 $${\text{R}}\alpha = {\text{y}}^{ + } - {\text{y}}^{ - }$$

7 $${\text{y}}_{{\text{u}}} = {\text{y}}_{{\text{u}}}^{ + } - {\text{y}}_{{\text{u}}}^{ - } = 0,\forall {\text{u}} \in {\text{U}}$$

8 $$\alpha^{\min } \le \alpha \le \alpha^{\max }$$

9 $$\alpha_{{\text{j}}}^{{{\text{min}}}} = \left\{ {\begin{array}{*{20}c} {1,{\text{ if s}}_{{{\text{uj}}}} \ne 0} \\ {0,{\text{ otherwise}}} \\ \end{array} } \right.,\quad { }1 \le {\text{j}} \le {\text{n}}.$$

The formulation in Eq. (5) corresponds to minimizing the sum of absolute values of stoichiometric coefficients of the lumped reaction, implemented by a well-established transformation of variables^31^. We make sure that every reaction involving the metabolite $u \in U$ (of unknown ${\Delta }_{f} G^{0}$), which we aim to eliminate, is associated a positive coefficient in the linear combination (Eqs. (8) and (9)). The reactions that are combined in the lumped reaction are given by the support of $\alpha$, with the difference of $y^{ + }$ and $y^{ - }$ denoting the stoichiometric coefficients of the lumped reaction (Eqs. (6) and (7)). Clearly, the lumping program can be iteratively applied for each metabolite with unknown ${\Delta }_{f} G^{0}$ (i.e. |U|= 1), which we refer to as naïve iterative procedure, or for a group of metabolites with unknown ${\Delta }G_{f}$ (i.e. |U|> 1).

The set *R* of reactions to be lumped is specified by the user. In the tests we conduct, we exclude biomass and exchange reactions from *R* since the biomass reaction is synthetic and the exchange reactions are often poorly supported with evidence. Exclusion of the biomass reaction from the set *R* was done to prevent an infeasible lumped reaction that would lead to blocking of the biomass reaction (see constraint in Eq. (2), for $y_{k} = 0$). In the provided implementation, we include the option to enable or disable lumping of (internal) transport reactions. The presented result allow lumping of transport reactions, whereby adjustment for transport across membrane is performed for lumped reactions that cross compartments.

### Group implementation

With the group implementation, we aim to eliminate all metabolites in a group at once by checking if the stoichiometric coefficients for the metabolites in the group can be set to zero by a linear combination that satisfies the constraint in Eqs. (6)–(9). If this is possible, a single linear program suffices to find a lumped reaction that eliminates the metabolites in the group (Fig. 1b—blue part). An example where the group procedure can be applied is given by the group U1 on Fig. 1a (bottom). Here, the lumping of ${{\varvec{r}}}_{1}$, ${{\varvec{r}}}_{2}$, and ${{\varvec{r}}}_{7}$ simultaneously eliminates the metabolites A and B, forming U1, by solving a single linear program.

The group lumping fails to find a lumped reaction if the feasible space of the linear program is empty, i.e. there exists no linear combination that can eliminate the metabolites with unknown ${\Delta }_{f}{G}^{0}$ in the group $U$ (Fig. 1b). If this is the case, we proceed with sequential lumping. For instance, the group U2, of metabolites E, G, and H, cannot be eliminated at once using the group implementation. The reason is that the linear system:

$$\left[ {\begin{array}{*{20}c} { - 1} & 1 & { - 1} & 0 \\ 0 & { - 1} & 0 & 1 \\ { - 1} & 0 & 1 & { - 1} \\ \end{array} } \right]*\left[ {\begin{array}{*{20}c} {\alpha _{3} } \\ {\alpha _{4} } \\ {\alpha _{5} } \\ {\alpha _{6} } \\ \end{array} } \right] = \left[ {\begin{array}{*{20}c} 0 \\ 0 \\ 0 \\ \end{array} } \right]$$

does not have a solution which satisfies the constraint that ${\alpha }_{3}$ is of value at least 1 since ${r}_{3}$ contains a metabolite from the group U2. However, metabolite G (corresponding to the second row) can be eliminated by using the same linear system, by a linear combination of reactions ${r}_{4}$, ${r}_{5}$, and ${r}_{6}$.

### Sequential implementation

The sequential implementation starts each iteration with a single metabolite ${\varvec{u}}$ with unknown ${{\varvec{\Delta}}}_{{\varvec{f}}}{{\varvec{G}}}^{0}$ from a given group ${\varvec{U}}$ of such metabolites. Here, we form a subset of metabolites with unknown ${{\varvec{\Delta}}}_{{\varvec{f}}}{{\varvec{G}}}^{0}$, denoted by ${{\varvec{U}}}^{{{\prime}}}$ which initially contains only ${\varvec{u}}$. The sequential implementation then aims to identify a linear combination of reactions that eliminates all metabolites in ${{\varvec{U}}}^{{{\prime}}}$, which is updated iteratively. If such a linear combination exists, the sequential implementation checks if the lumped reaction involves any other metabolite, say ${\varvec{w}}$, of unknown ${{\varvec{\Delta}}}_{{\varvec{f}}}{{\varvec{G}}}^{0}$. In such a case, the found lumped reaction ${\varvec{y}}$ is added to the matrix ${\varvec{R}}$ and metabolite ${\varvec{w}}$ is added to the set ${\varvec{U}}^{\prime}$. Clearly, then, we need to reiterate the solving of the linear program to ensure that all metabolites in the updated set ${\varvec{U}}^{\prime}$ are eliminated (Fig. 1b—magenta part).

Note that in solving the linear program in Eqs. (5)–(9), we do not consider the existence of several alternative solutions, to prevent backtracking. This is justified by our aim to eliminate a maximal rather than the maximum number of metabolites with unknown ${\Delta }_{f} G^{0}$ via reaction lumping. However, this may have implications in constraints in TMFA applications. The sequential implementation necessitates solving of several linear programs with an increasing size of $R$. The latter is due to the fact that in each iteration $R$ is extended with the currently found solution. For instance, let us consider group U2 consisting of three metabolites that cannot be eliminated by the group implementation. The sequential solution procedure would first search for a linear combination that eliminates metabolite *E*. While the linear combination *y* = $r_{3} + 2r_{4} + r_{5}$ removes metabolite *E*, it also involves metabolite $G$ which is also of unknown ${\Delta }_{f} G^{0}$. Thus, the set $U^{\prime}$ is enlarged to now include metabolite $E$ and $G$ and the matrix $R$ is augmented with the found solution $y$. The solution to the next linear program identified $y + 2r_{6}$ as a linear combination that eliminates metabolite $G$; however, the solution includes the metabolite $H$ of unknown ${\Delta }_{f} G^{0}$. The final iteration is performed with the set $U^{\prime} = \left\{ {E,G,H} \right\}$ and the matrix $R$ enlarged again by previously found linear combination; however, no solution that eliminates all three metabolites in $U^{\prime}$ can be identified in this case. Proceeding with metabolite $G$, two iterations are needed to identify the lumped reaction, $r_{4} + r_{5} + r_{6} ,$ that eliminates *G*. Similarly to *E*, no linear combination eliminates metabolite *H* while constraining metabolite $E$ and $G$ to zero which are added to $U^{\prime}$ in the proceedings to find a lumped reaction. In total eight linear programs need to be solved to exhaustively check if any metabolites of group U2 can be eliminated. In contrast to the group implementation, the sequential implementation is capable to identify a lumped reaction that eliminates the metabolite $G$ with unknown ${\Delta }G_{f}$. Furthermore, in contrast to the group implementation, which eliminates the group U1 in a single linear program, the sequential implementation requires solving four linear programs to arrive at the same solution.

### Combined procedure

The combined implementation applies first the group and then sequential implementation on each of the groups of metabolites resulting from the partition. Therefore, it ensures maximizing the number of metabolites with unknown ${\varvec{\Delta}}{{\varvec{G}}}_{{\varvec{f}}}$ that can be eliminated via lumping while solving a fewer number of linear programs (Fig. 1b). In effect, this approach takes the advantages of the speed of the group implementation, due to the reduced number of programs to be solved, and the exhaustive search of the sequential lumping.

### TMFA and variability analysis

We implemented TMFA by allowing the concentrations to range between 1 µM and 20 mM. In the case of *E. coli* the range for Glycerophosphoglycerol (g3pg), Sn-Glycero-3-phosphoethanolamine (g3pe), and water in the cytosol had to be relaxed to 1 µM –$1.4*{10}^{55}$ M, 1 µM-6003 M and 14.92 pM-20 mM, respectively, for both the original model and the model with lumped reactions. Additionally, for the model with lumping, the upper boundary of Nicotinamide adeneine dinucleotide (nadh) had to be expanded to 41.2 mM to obtain 90% of optimal biomass from FBA. Note that similar relaxations need to be performed in applications of TMFA with other models^18,32^*.* We then determined the variability of $\Delta {G}_{j}$ for every reaction $j$, by calculating the minimum and maximum values it takes in the original model and the model with the lumped reactions at 90% of the optimal biomass from FBA.

### Technical details of the implementation

The lumping procedure was implemented in MATLAB (v 9.6.0^33^), requiring MATLAB’s solver ‘intlinprog’ as it outperformed other solvers within a benchmark showcase^34^. All options in the solver were left to default apart from ‘MaxTime’ which was set to 120 s (which is roughly 10 times greater than the longest duration observed in all investigated cases). The time limit had no influence on the outcome as it never caused a premature stop. The statistical analysis was conducted in R (v. 4.0.2) requiring the packages ggplot2^35^, ggnewscale^36^, gridExtra^37^, RColorBrewer^38^, mdthemes^39^ and cowplot^40^. Figures 1 and 3 were created using LucidChart^41^. All computations were done on a Desktop PC with AMD Ryzen 5 processor (6 × 3.60 GHz) and 32 GB DDR4 RAM.

## Results

Our proposed lumping procedure is designed to identify lumped reactions for any model with incomplete thermodynamic data to enforce stricter thermodynamic constraints, see Eqs. (1)–(4). As stated above, the existing applications of TMFA^11,18^ do not specify how to systematically find such combination of reactions that lead to elimination of metabolites of unknown ${\Delta }_{f}{G}^{0}$. The proposed combined procedure can be applied to genome-scale models, independent of the extent of available information and its output can be directly used with existing implementation of TMFA^42^.

### Lumping decreases the number of reactions with undetermined ${\varvec{\Delta}}{{\varvec{G}}}^{0}$

We applied the combined lumping procedure with the genome-scale models of three organisms *E. coli*^43^, *B. subtillis*^44^_,_ and *H. sapiens*^27^. For every model, each reversible reaction is split into two irreversible reactions. The genome-scale model *E. coli* iJR904 contains 1303 reactions and 756 metabolites of which 38 and 26 are not annotated with ${\varvec{\Delta}}{{\varvec{G}}}^{0}$ and ${{\varvec{\Delta}}}_{{\varvec{f}}}{{\varvec{G}}}^{0}$, respectively. This model was selected since it represents one of the most complete models regarding thermodynamic data. Lumping is applied on the reduced matrix of size 73 metabolites and 32 reactions. Note, that matrix ${\varvec{R}}$ does not include the biomass reaction and exchange reactions (“Methods”). The complete set of metabolites without any ${{\varvec{\Delta}}}_{{\varvec{f}}}{{\varvec{G}}}^{0}$ information was partitioned into 12 groups in an automated fashion, based on joint occurrence in at least on reaction (“Methods”). The model of *B. subtilis* iBsu1103 contains 2774 reactions and 1381 metabolites, of which 2025 and 434 are of unknown ${\varvec{\Delta}}{{\varvec{G}}}^{0}$ and ${{\varvec{\Delta}}}_{{\varvec{f}}}{{\varvec{G}}}^{0}$, respectively. This is a representative of a model which includes little thermodynamic data. Lumping was applied on the reduced matrix of dimension 1346 × 1971. The complete set of metabolites with unknown ${{\varvec{\Delta}}}_{{\varvec{f}}}{{\varvec{G}}}^{0}$ was divided into 7 groups in an automated fashion (“Methods”). A recent model of *H. sapiens* redHuman compromises 11,139 reactions and 4521 metabolites of which 3145 and 1032 are of unknown ${\varvec{\Delta}}{{\varvec{G}}}^{0}$ and ${{\varvec{\Delta}}}_{{\varvec{f}}}{{\varvec{G}}}^{0}$, respectively. The model was selected to test the effect of model size on the performance of the procedure. All metabolites with unknown ${{\varvec{\Delta}}}_{{\varvec{f}}}{\varvec{G}}$ are split into 280 groups by following our implementation (“Methods”).

**Figure 2:**
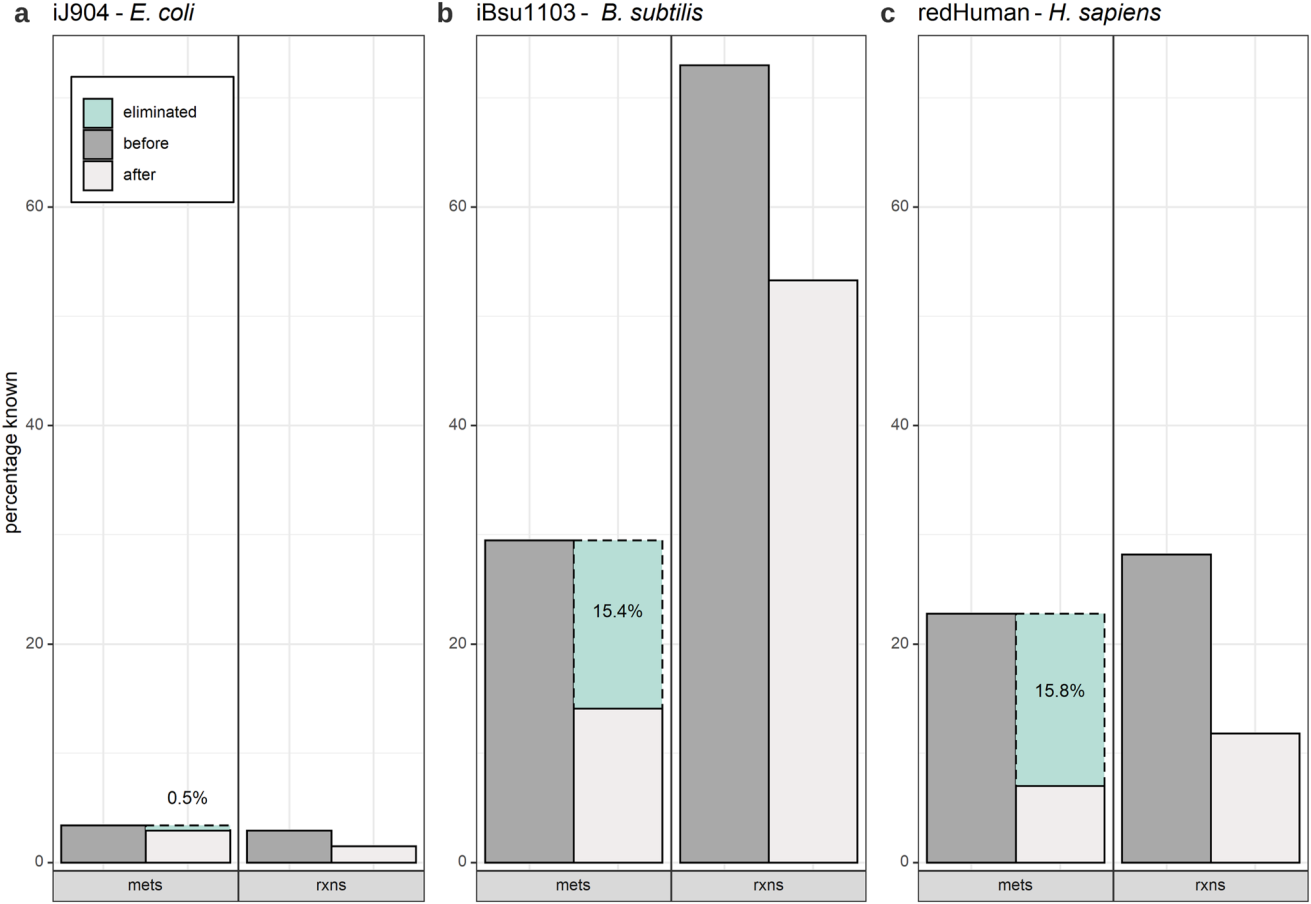
Application of lumping on genome-scale metabolic models. The percentage of reactions with unknown $\user2{\Delta G}^{0}$ is in three GEMs, (**a**) iJR904, (**b**) iBsu1103, and (**c**) redHuman, reduced after lumping due to elimination of metabolites with unknown ${\varvec{\varDelta}}_{{\varvec{f}}} {\varvec{G}}^{0}$. Shown is the percentage of unknown $\user2{\Delta G}$-values in relation to the total number of present metabolites (mets) and reactions, respectively, (rxns) before and after the lumping procedure was applied. The number of eliminated metabolites with unknown ${\varvec{\varDelta}}_{{\varvec{f}}} {\varvec{G}}^{0}$ is with respect to the total number of metabolites. The investigated genome-scale models have low, high and medium number of unknown $\user2{\Delta G}^{0}$ and ${\varvec{\varDelta}}_{{\varvec{f}}} {\varvec{G}}^{0}$, respectively, and pertain to organisms of increasing complexity.

**Figure 3:**
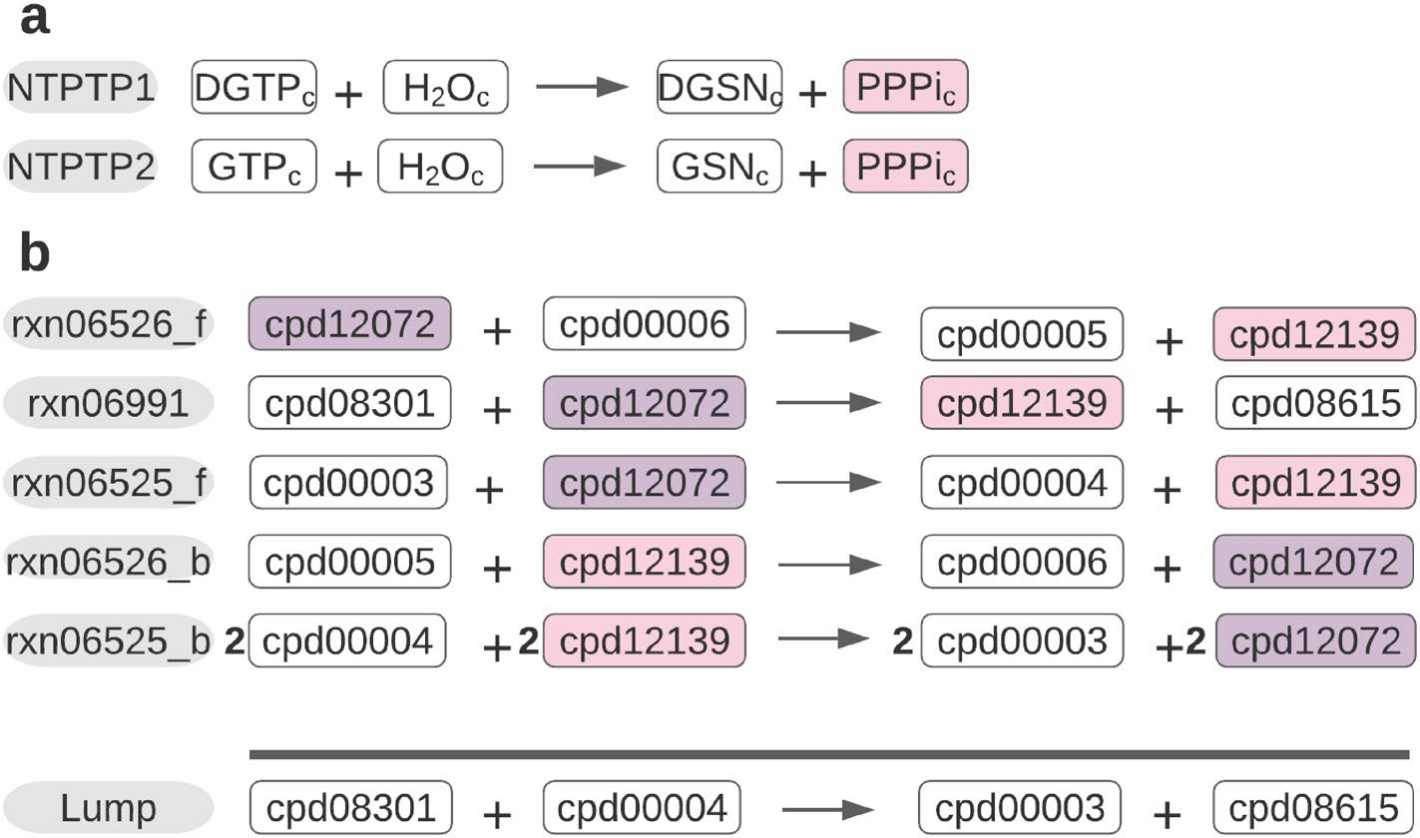
Real world examples of failure and success of lumping. An example for (**a**) failure taken from the iJ906 model and (**b**) success taken from the iBsu1103 model of proposed lumping procedure is shown. Colored metabolites indicate the respective missing ${\varvec{\varDelta}}_{{\varvec{f}}} {\varvec{G}}^{0}$ and hence target of lumping. Within each example the same color corresponds to the same metabolite. Bold numbers indicate a multiplicative coefficient.

By applying the proposed lumping procedure, we were able to investigate the extent to which metabolites with unknown ${\Delta }_{f} G^{0}$ could be removed, thus leading to more reactions with specified ${\Delta }G^{0}$. For all three models we could eliminate a sizeable proportion of metabolites with unknown ${\Delta }_{f} G^{0}$ (Fig. 2). In the iJR904 model, elimination of 0.5% metabolites (with unknown ${\Delta }_{f} G^{0}$) led to a decrease of 1.4% of reactions with unknown ${\Delta }G^{0}$(the percentages are with respect to the total number of metabolites and reactions). The decrease was not due to being able to estimate the unknown ${\Delta }G^{0}$, but rather to the inclusion of lumped reactions in the model for which ${\Delta }G^{0}$ could be readily determined from the provided thermodynamics data. For instance, inorganic triophosphate (PPPI) cannot be removed, by lumping, from the cytosol due to the reactions in which it occurs (Fig. 3a). In the model of iBsu1103, 15.4% of metabolites (with unknown ${\Delta }_{f} G^{0}$) could be eliminated, leading to a decrease of 19.7% for the reactions with unspecified ${\Delta }G^{0}$. Therefore, our procedure eliminated more than half of the metabolites with unknown ${\Delta }_{f} G^{0}$. For example, two metabolites with unknown ${\Delta }_{f} G^{0}$ can be removed from this model by lumping five reactions (Fig. 3b). Similarly, for the redHuman model, 15.4% of metabolites with unknown ${\Delta }_{f} G^{0}$ could be eliminated, leading to a decrease of 16.4% in reactions with known ${\Delta }G^{0}$. Thus, irrespective of the relative amount of available information regarding ${\Delta }_{f} G^{0}$, our proposed lumping procedure identified combinations of reactions with unidentified ${\Delta }G^{0}$ which eliminates the maximal number of metabolites with unknown ${\Delta }G_{f}$.

### Combined procedure is more efficient in comparison to the sequential implementation alone

A lumping procedure that naively attempts to eliminate every metabolite of unknown ${{\varvec{\Delta}}}_{{\varvec{f}}} {\varvec{G}}^{0}$ would rely solely on the iterative procedure. This approach would require solving at least one linear program per metabolite of unknown ${{\varvec{\Delta}}}_{{\varvec{f}}} {\varvec{G}}^{0}$ to identify a lumped reaction that eliminates the metabolite. The novelty of our solution consists of reducing the number of linear programs to be solved, by proposing the combined procedure that relies on the group and sequential implementation executed on each group of metabolites (see “Methods”). The group implementation has the potential to speed up the elimination of the metabolites in a group by solving a single linear program. Here, we first investigated the advantages provided by the combined procedure measured by the difference between the total duration and number of linear programs solved in comparison to the naïve usage of the sequential procedure. We also compared the time spent on solving the linear programs within the group and sequential component of the combined procedure (Table 1).

**Table 1:** Comparison of the combined and sequential implementation.

	No. LPs	Total time (s)	Min time of LP (s)	Max time of LP (s)
**iBsu1103**				
Sequential only	1003	179	0.12	0.33
Combined	1006	196	0.09	14.31
**redHuman**				
Sequential only	4500	2005	0.19	0.90
Combined	2650	1233	0.19	13.59

The comparison is carried out on two genome-scale models, iBsu1103 and redHuman. Shown are the number of linear programs (LPs), total time for the execution of the procedure, and the minimum and the maximum time (in seconds) needed to solve a single linear program within the entire procedure.

The combined procedure for the iBsu1103 model required solving 1006 linear program in 196 s. The linear program which took the least amount of time required 0.09 s, while the slowest required 14.31 s (Table 1). The lumping based on the sequential implementation only necessitated solving of three linear program fewer in comparison to the combined procedure, the duration of which ranges from 0.12 to 0.33 s. The entire procedure relying on the sequential implementation took only 179 s and was faster by 17 s in comparison to the combined (Table 2).

**Table 2:** Properties of the investigated genome-scale metabolic models.

Model	No. metabolites (unknown ${\Delta }_{f} G^{0}$)	No. reactions (unknown ${\Delta }G^{0}$)	R dimension	No. groups
iJ906	756 (26)	1303 (38)	73 × 32	12
iBsu1103	1381 (434)	2774 (2025)	1346 × 1971	7
redHuman	4521 (1032)	11,139 (3145)	1993 × 3016	280

Shown are the number of metabolites and the number of those with unknown ${\varvec{\varDelta}}_{{\varvec{f}}} {\varvec{G}}^{0} ,$ the number of reactions and the number of those with unknown $\user2{\Delta G}^{0}$, the dimensions of the matrix ${\varvec{R}}$ of reactions used in the lumping procedure, and the number of metabolite groups.

Despite the better performance (in time) for the sequential procedure on the iBsu1103 model, the potential of the combined implementation was demonstrated on the case of the redHuman model. Here, the combined procedure required solving 2650 linear programs in 2659 s. The fastest linear program took 0.19 s, while the slowest required 13.59 s. The lumping based only on the sequential procedure required 4500 linear programs, with time that ranged from 0.19 to 0.9 s. In comparison to the combined procedure, the application of only the sequential took 772 s longer and required solving 1850 more linear programs (Table 2).

**Figure 4:**
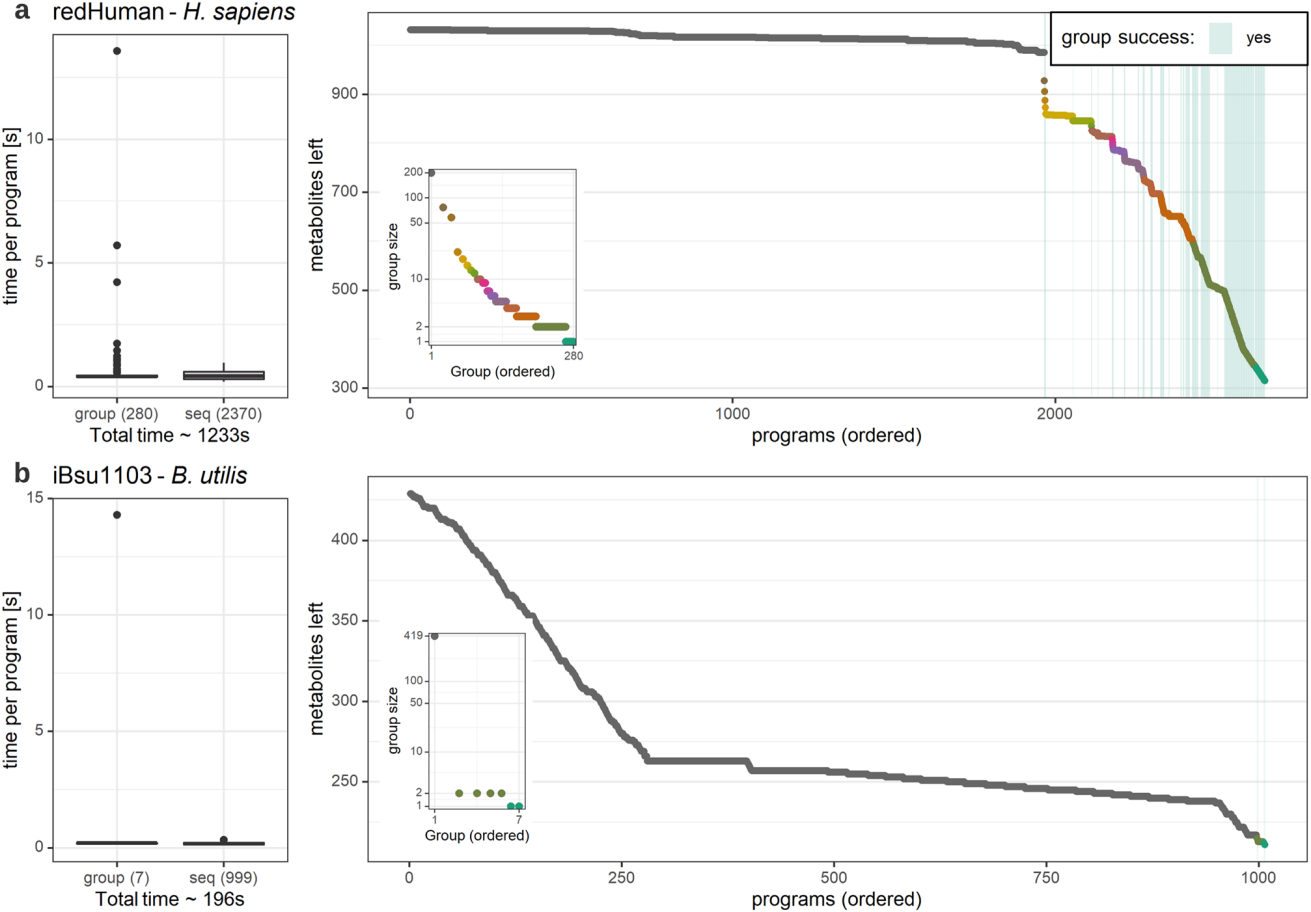
Analysis of time efficiency of the proposed combined procedure. The inclusion of the group procedure in the combined lumping procedure is capable to remove a group of metabolites with solving only one linear program instead of a succession of at least one program per metabolite in the group. Boxplots show the distribution of time needed per linear program separated into the group and sequential procedure with outlier (dots) displayed if the value lies beyond 1.5*IQR of respective quantile. The insets show the number of metabolites in respective group—the colours matching those in the bigger graph, which displays the progress of the lumping procedure. (**a**) During the lumping of redHuman 280 group and 2041 sequential programs were needed. The groups are ordered and coloured by their group size. If a group-program can find a solution all metabolites in respective group are removed, resulting in a sudden drop, marked here by light green lines (241 successful). Otherwise, the group program was followed by the sequential program for each metabolite in the group. (**b**) During the lumping of iBsu 7 group (4 successful) and 888 sequential programs were needed. Here, the majority of metabolites form a single group, for which respective LP does not find a solution, leading to a stepwise decrease in the number of removed metabolites.

Next, we investigated the contribution of the group and sequential implementation to the combined procedure. The proposed combined lumping procedure required 280 and 2370 linear programs for the group and the sequential implementation parts, respectively, taking all together ~ 20 min (Fig. 4a). An overall trend is apparent that, in most cases, the fewer linear programs for the group implementation took longer than the linear programs for individual linear programs in the sequential approach (Fig. 4a). The most prominent outliers in terms of the time needed to solve the linear programs corresponded to the largest groups, of size 200 for the redHuman model and of size 419 for the iBsu1103 model, taking 14.31 and 13.59 s, respectively. For the redHuman model, the group implementation was infeasible for the largest group, but succeeds to lump groups of sizes 77 and 58. This led to the removal of 135 metabolites with unknown ${\Delta }_{f} G^{0}$ by solving only two linear programs, one for each of the two groups. In addition, the number of groups to which the group implementation yields a solution increases with the decreasing size of the groups.

The iBsu1103 model has a higher degree of missing thermodynamic data, leading to groups of sizes as large as 419 (out of 434) metabolites with missing ${\Delta }_{f} G^{0}$ (Fig. 4b). As only seven groups were found, the trend of the group implementation taking longer to calculate was not as striking as for the redHuman model, but was still present with respect to the median time (0.199 vs 0.162). The group implementation was not feasible for the largest group, but the corresponding linear program took 13.59 s to solve. As a result, for each metabolite in this group, the sequential implementation had to be employed. The metabolites with unknown ${\Delta }_{f} G$ are eliminated stepwise compared to the sudden drop, displayed in the redHuman model (Fig. 4b). The linear programs for the group implementation in the case of the smaller, remaining groups are feasible (Fig. 4b). As there were only seven groups present, no reliable trend regarding group size and success could be further established.

The application of the combined procedure on the two models demonstrated that the group implementation provides speed-up due to a reduced number of linear programs to solve. In addition, our results showed that the group procedure adds marginal increase in running time, in case it needs to be followed by the sequential procedure.

### Benefits of the lumping procedure in TMFA applications

To demonstrate the advantages of using the models that consider lumped reactions, based on the proposed procedures, we implemented TMFA for the models of *E. coli* and *B. subtilis* (see “Methods”). We then computed the range of values that $\Delta G_{j}$ takes for every reaction $j$ in the original model and the model with lumped reactions at the optimal biomass obtained from TMFA. This analysis allowed us to classify the reactions into irreversible and reversible based on the sign that the maximum $\Delta G_{j}$ takes. The reversible reactions could further be divided into those whose range is reduced upon lumping and those whose ranges show shifts between the two models.

In the case of *E. coli*’s model, we found that the lumping changed the number of irreversible reactions from 353 in the original model to 350 in the one that also considered the lumped reactions to impose thermodynamic constraints (Table 3). As a result, the number of reversible reaction was reduced from 707 in the original to 704 in the model with lumped reactions (Table 3). The reactions deemed irreversible in the *E. coli* model with lumped reactions include: UAG2E, DAPabc, and ACMAMUT (see Supplementary Figure S1). Expectedly, the consideration of lumped reactions decreased the ranges of 116 reversible reactions (see Supplementary Figure S2).

**Table 3:** Distribution of reversible/irreversible reactions with and without lumping.

Model	No. irreversible reactions		No reversible reactions	
	Without lumping	With lumping	Without lumping	With lumping
iJ906	350	353	707	704
iBsu1103	3	3	1678	1678

Shown are the numbers or reversible and irreversible reactions for the models iJ906 and iBsu1103 with and without lumping. A reaction is defined as irreversible if respective $\user2{\Delta G}$-range is strictly negative, which is determined with a variability analysis ensuring optimal biomass.

In the case of *B. subtilis*’ model, we observed that there is no change in the number of irreversible and reversible reactions with and without consideration of lumped reaction (Table 3). However, the ranges of Gibbs free energy were substantially reduced for 101 reactions (see Supplementary Figure S3). Therefore, we conclude that the lumping procedure does have an effect on the findings from TMFA and can be effectively used to obtain more constrained predictions, particularly for metabolite concentrations (that are interlinked with the values of Gibbs free energy).

## Discussion

The idea of reaction lumping has been introduced to provide additional constraints for reactions with unknown ${\Delta }G^{0}$, but can be linearly combined into an overall lumped reaction whose ${\Delta }G^{0}$ can be easily determined based on the thermodynamic data for the modelled components. The applications of TMFA are in part hampered by the lack of good coverage of thermodynamic data^19,32,45^, leaving the flux solution space less constrained. Here, we proposed an algorithm which fills the gap between the introduction of the idea of reaction lumping and the TMFA approach that imposes special constraints to the lumped reactions. Our procedure starts with the identification of groups of metabolites with unknown ${\Delta }_{f} G^{0}$, identified by inspecting the connected components of the bipartite metabolite reaction graph. The identification of lumping reactions can thereby be solved independently on each of the groups using the combined lumping procedure. The combined lumping procedure consists of solving one linear program, for the group implementation, and a series of linear programs that are iteratively solved, for the iterative implementation. The group component can be interpreted as a shortcut, by testing whether all metabolites in a group can be eliminated at once. In addition, the proposed combined procedure maximizes the number of metabolites with unknown ${\Delta }_{f} G$ that can be eliminated via reaction lumping.

In general, the linear program in the group implementation takes a longer time to solve due to the larger number of constraints it includes. Although the sequential procedure starts with smaller linear programs, requiring shorter time to be solved, the iterative process leads to an increasing number of such program that include a larger number of both constraints and variables. The total duration, thus, depends on the particularities of the analyzed model. In our comparative analysis we presented a case when the combined procedure performs worse than the naive sequential implementation applied alone. In this case, only few groups of metabolites with unknown ${\Delta }_{f} G^{0}$ were found, including a dominant group that includes almost all such metabolites. The situation differs in the scenario where the groups of metabolites with unknown ${\Delta }G_{f}$ are more evenly distributed, leading to the advantages of the combined procedure.

The recently published toolbox to conduct TMFA, called matTFA, states that any reaction involving metabolites with unknown ${\Delta }_{f} G^{0}$ “will not be constrained with thermodynamics”^42^. Our proposed group lumping procedure investigates whether such reactions can be constrained with thermodynamics. The shown decrease of reactions with unknown ${{ \Delta }}G^{0}$, with respect to the total number of reactions, corresponds to a gain in constraints which has the potential to further restrict the solution space without the need of any additional data.

Our comparative analysis on three genome-scale models that differ in terms of size and complexity of the modelled metabolic pathways shows that the lumping procedure is time-efficient, systematic, and results on reproducible findings. The proposed combined procedure clearly defines how lumped reactions are formed and ensures that ${\Delta }G^{0}$ can be calculated for each lumped reaction. In contrast, previous studies only provide a list of lumped reactions, without specifying how they were obtained, thus not ensuring reproducibility^18^. The reproducibility of our findings is further guaranteed by the provided implementation of the proposed procedure and accessibility of all data, following the FAIR principles. The implementation is general to allow the identification of lumped reactions in any metabolic model complying with the input specifications. Therefore, the systematic way for identification of lumped reactions by the proposed combined procedure has the potential to further propel the applications of TMFA, due to the increasing availability of quantitative metabolomics data^46^. Indeed, our application of TMFA with and without consideration of lumped reactions in two genome-scale models shows that the lumping procedure provides for more constrained predictions of metabolic phenotypes.

The provided procedure allows some flexibility with respect to the choice of reactions to be lumped. In our analysis, we did not consider synthetic and exchange reactions in the lumping, and future efforts will be dedicated to the consideration of these cases. In addition, follow-up studies will be dedicated to investigative the effect of alternative solution in the detection of lumped reactions on the possibility to maximize the number or metabolites with unknown ${\Delta }_{f} G^{0}$ that can be eliminated by the proposed combined procedure.

## Supplementary Information

Supplementary Information

## Data Availability

All data and source code are available at: https://github.com/LeaSeep/ReactionLumping.
