# Supplementary material for: Reaction lumping in metabolic networks for application with thermodynamic metabolic flux analysis
Source: Sci Rep. 2021 Apr 20;11:8544. doi: 10.1038/s41598-021-87643-8 (PMC8058346; doi:10.1038/s41598-021-87643-8)
Supplement: Supplementary file 1 — Supplementary Information [file 41598_2021_87643_MOESM1_ESM.pdf]

Reaction lumping in metabolic networks for application with thermodynamic metabolic flux analysis- Supplementary

Lea Seep, Zahra Razaghi-Moghadam and Zoran Nikoloski

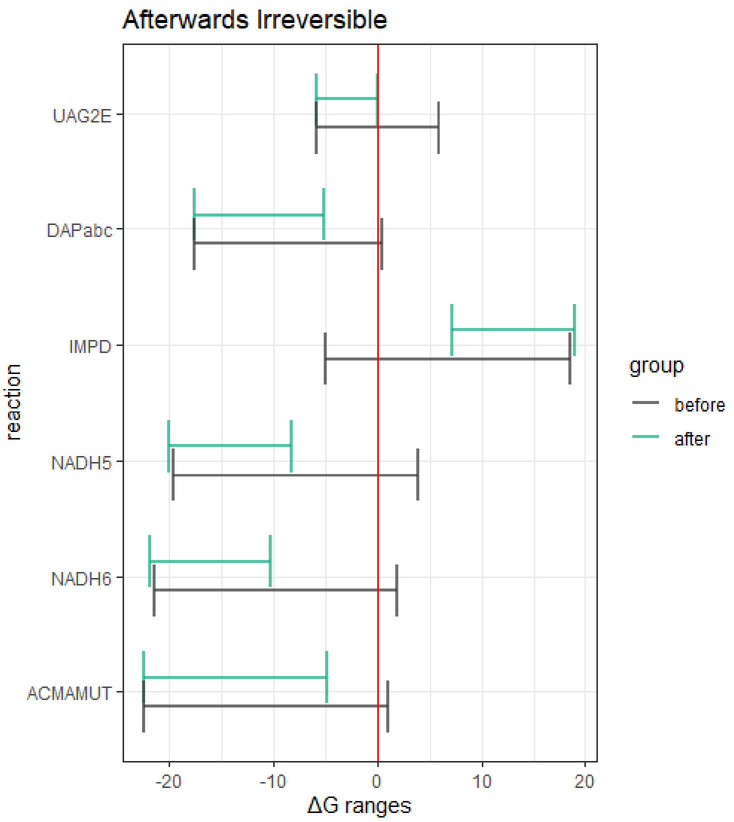

Figure S1 – Reactions deemed irreversible after lumping in *E. coli*. The ΔG-ranges of all reactions that can be only determined irreversible after lumping. Grey coloured bars show the range before, green coloured bars after lumping is applied.

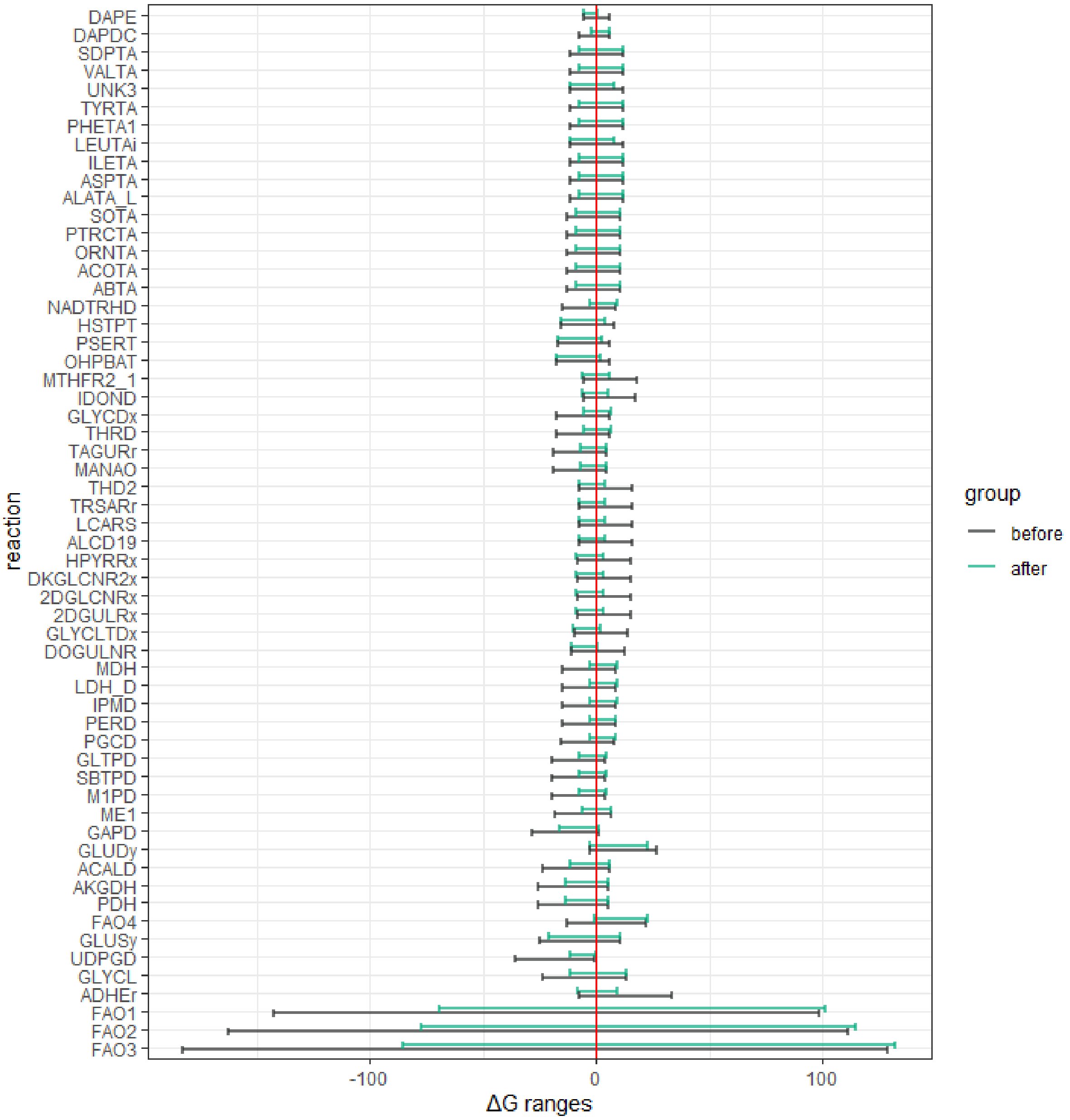

Figure S2 – Decreased ΔG-ranges after lumping in *E. coli*. All reactions that show a decrease regarding the variable space of ΔG after lumping is applied. The grey bars indicate the range before, the green bars after lumping was applied.

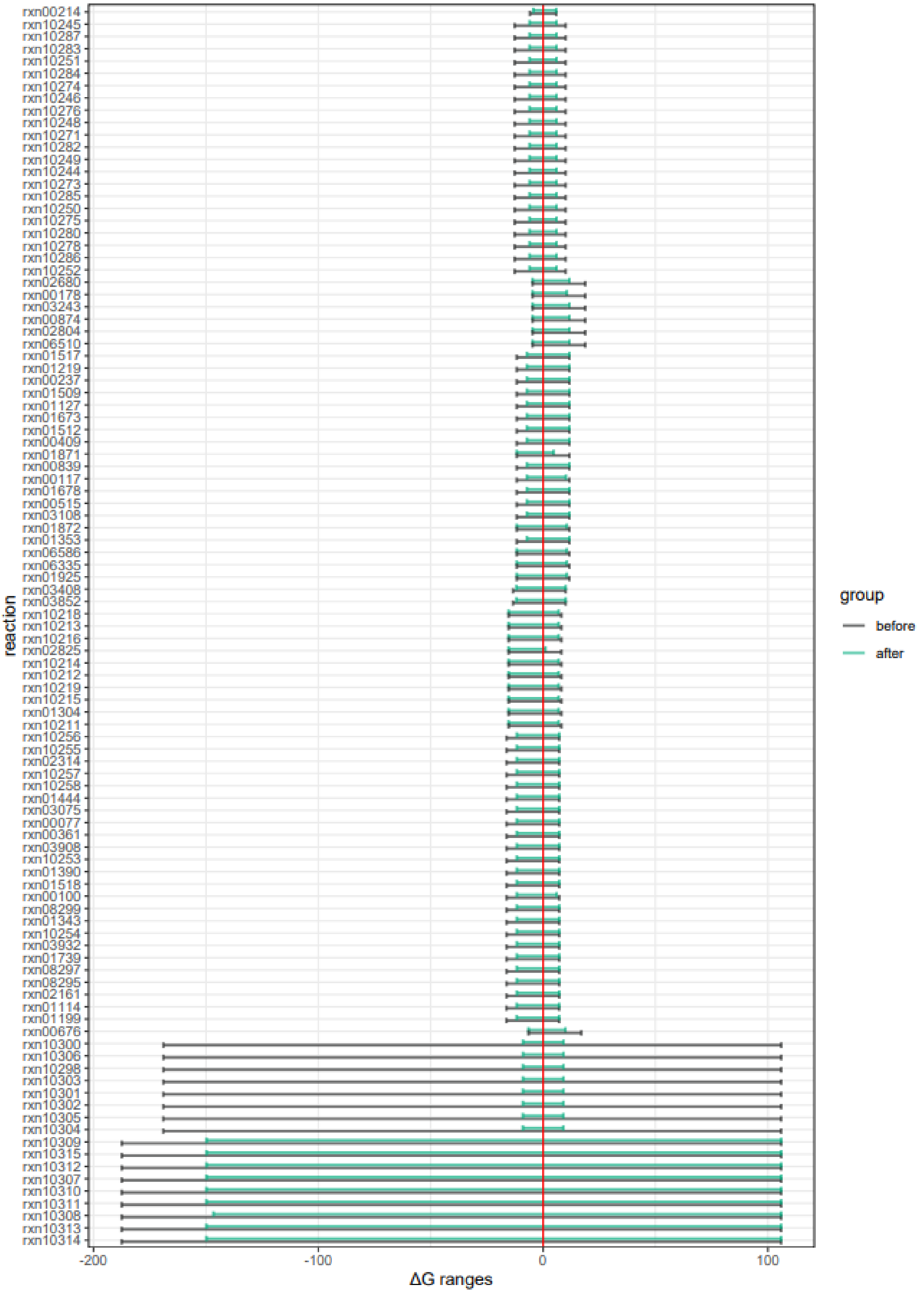

Figure S3. Different ΔG-ranges after lumping in *B. subtilis*. All reactions that exhibit either a change or a shift of respective ΔG-range after lumping is applied. Grey coloured bars show the range before, green coloured bars after lumping is applied.
